# Functional MRI radiomics-based assessment of pelvic bone marrow changes after concurrent chemoradiotherapy for cervical cancer

**DOI:** 10.1186/s12885-022-10254-7

**Published:** 2022-11-08

**Authors:** Xiaohang Qin, Cong Wang, Guanzhong Gong, Lizhen Wang, Ya Su, Yong Yin

**Affiliations:** 1 Department of Graduate, Shandong First Medical University, Shandong Academy of Medical Sciences, Jinan, China; 2 Department of Radiation Physics, Shandong Cancer Hospital and Institute, Shandong First Medical University and Shandong Academy of Medical Sciences, Ji Yan Road No.440, 250117 Jinan, China; 3 Department of the Fourth Ward of Gynecologic Oncology, Shandong Cancer Hospital and Institute, Shandong First Medical University, Shandong Academy of Medical Sciences, Jinan, China

**Keywords:** Radiomics features, IDEAL IQ, Bone marrow sparing, Cervical cancer, Hematologic toxicity

## Abstract

**Objectives:**

To quantify the dose-response relationship of changes in pelvic bone marrow (PBM) functional MR radiomic features (RF) during concurrent chemoradiotherapy (CCRT) for patients with cervical cancer and establish the correlation with hematologic toxicity to provide a basis for PBM sparing.

**Methods:**

A total of 54 cervical cancer patients who received CCRT were studied retrospectively. Patients underwent MRI IDEAL IQ and T2 fat suppression (T2fs) scanning pre- and post-CCRT. The PBM RFs were extracted from each region of interest at dose gradients of 5–10 Gy, 10–15 Gy, 15–20 Gy, 20–30 Gy, 30–40 Gy, 40–50 Gy, and > 50 Gy, and changes in peripheral blood cell (PBC) counts during radiotherapy were assessed. The dose-response relationship of RF changes and their correlation with PBC changes were investigated.

**Results:**

White blood cell, neutrophils (ANC) and lymphocyte counts during treatment were decreased by 49.4%, 41.4%, and 76.3%, respectively. Most firstorder features exhibited a significant dose-response relationship, particularly FatFrac IDEAL IQ, which had a maximum dose-response curve slope of 10.09, and WATER IDEAL IQ had a slope of − 7.93. The firstorder-Range in FAT IDEAL IQ and firstorder-10Percentile in T2fs, showed a significant correlation between the changes in ANC counts under the low dose gradient of 5–10 Gy (*r* = 0.744, -0.654, respectively, *p* < 0.05).

**Conclusion:**

Functional MR radiomics can detect microscopic changes in PBM at various dose gradients and provide an objective reference for bone marrow sparing and dose limitation in cervical cancer CCRT.

**Supplementary Information:**

The online version contains supplementary material available at 10.1186/s12885-022-10254-7.

## Introduction

Concurrent chemoradiotherapy (CCRT) is the standard treatment for locally advanced cervical cancer [1]. Compared to radiation therapy (RT) and chemotherapy alone, CCRT improves the overall survival rate and local control rate, with a 5-year survival rate of 30-80% [2, 3]. CCRT improved the prognosis of patients with cervical cancer, while the incidence of hematological toxicity (HT) of G2 + was 69.5% [1]. Due to severe bone marrow suppression, treatment may be delayed or discontinued [4, 5].

The bone marrow consists of red and yellow bone marrow with hematopoietic activity and high-fat content, respectively, which is highly sensitive to radiation. Irradiation can cause red-to-yellow bone marrow transformation, decreased bone marrow activity, and even irreversible injury. Studies have reported that the dose and volume of pelvic bone marrow (PBM) irradiation were significantly related to acute HT [6–8]. Patients with V10 ≥ 85% were more likely to develop HT (39% vs. 27%) in RTOG0418-based clinical trials [9]. Kumar [10] et al. discovered a link between G4 HT and PBM-V5 > 95% and V20 > 45%. The HT produced during CCRT will last for at least 3 months after treatment is completed [11].

Dose-volume parameter of bone marrow is the main index for predicting HT. However, the prediction indices and dose-volume parameters used in different studies varied, and no optimal radiation dose limitation for bone marrow were proposed. The primary reason for this is that bone marrow radiotherapy response is a dynamic process. The study of bone marrow radiotherapy response under various dose gradients can provide a foundation for accurate prediction of bone marrow dose limitation and damage.

The foundation for tracking the dynamic response and investigating bone marrow injury is accurate identification and segmentation of bone marrow. Magnetic resonance imaging (MRI) describes information to describe the anatomical, structural, and functional properties of soft tissues, which can be used to monitor the response of tumors and normal tissues to radiation during RT and the follow-up period [12]. Quantitative MRI of the fat fraction has been shown to be sensitive to changes in bone marrow fat content. The use of IDEAL IQ to identify active bone marrow for RT planning in patients undergoing pelvic CCRT can reduce the risk of HT [13]. Our previous study, which used the MR IDEAL IQ sequence to examine changes in PBM fat content during cervical cancer CCRT, discovered that PBM fat content increased with accumulated radiation dose. The fat fraction increased by 74.2% from 48.5% in the high-dose irradiated bone marrow area [14].

The analysis of MR IDEAL IQ images can help to improve the tracking and prediction of bone marrow radiotherapy response. Radiomics can characterize microstructures, perfusion, and metabolism by extracting complex quantitative features from clinical image data and quantitatively analyzing dynamic changes in tumor or normal tissue during treatment. Combining magnetic resonance imaging and radiomics techniques allows for quantifying spatial information via texture analysis, which can reveal and track microscopic changes in bone marrow damage during CCRT for cervical cancer [12, 15].

The objective of this study was to use multisequence MR radiomics to quantify changes in the bone marrow before and after CCRT in patients with cervical cancer and to examine the relationship between changes in radiomics features (RF) and peripheral blood cell (PBC) counts.

## Materials and methods

### Patient population and characteristics

A total of 54 patients diagnosed with stage IB to IVB cervical cancer by the International Federation of Gynecology and Obstetrics (FIGO) at Shandong Cancer Hospital between January 2019 and December 2020 were retrospectively selected for this study (Table 1). The median age of the patients was 51 years (range: 20–65 years). The patients with fractures, inflammation, and intramedullary hemorrhage were excluded. All patients underwent external beam radiotherapy with intensity modulated radiotherapy (IMRT) or volumetric modulated arc therapy (VMAT) and concurrent cisplatin chemotherapy.

**Table 1:** Patient characteristics

n (%) Total *n* = 54	
Age	
Median (years, median, range)	51 (20–65)
Pathology, n (%)	
SCC	44 (79%)
CAC	10 (21%)
FIGO stage, n (%)	
I	18 (33%)
II	14 (26%)
III	21 (39%)
IV	1 (2%)
Radiotherapy days	
Median (days, median, range)	36 (31–45)
PTV prescription dose	
Median (Gy, median, range)	50 (45-50.4)
WBC at pre-RT (×10^9/L, median, range)	6 (3.19–9.52)
ANC at pre-RT (×10^9/L, median, range)	4 (1.72–8.21)
ALC at pre-RT (×10^9/L, median, range)	1.4 (0.53–2.74)
PLT at pre-RT (×10^9/L, median, range)	244 (113–471)
HGB at pre-RT (g/L, median, range)	120 (87–144)

*SCC *Cervical squamous cell carcinoma, *CAC *Cervical adenocarcinoma, *GTV *Gross tumor volume, *FIGO *International Federation of Gynecology and Obstetrics, *pre-RT *Before radiotherapy, *WBC *White blood cell, *ANC *Absolute neutrophil count, *ALC *Absolute lymphocytes count, *PLT *Platelets, *HGB *Hemoglobin

### Computed tomography (CT) simulation

A Philips 16-slice Brilliance big-bore CT scanner (Philips Medical Systems, Amsterdam, The Netherlands) was used for imaging. The patients were immobilized with a thermoplastic mold in a supine position or immobilized with an abdominal pelvic fixator in a prone position. Scanning was performed from the upper border of the T2 vertebra to the middle of the femur with a slice thickness of 3 mm and a slice gap of 3 mm.

### MR simulation

**Fig. 1:**
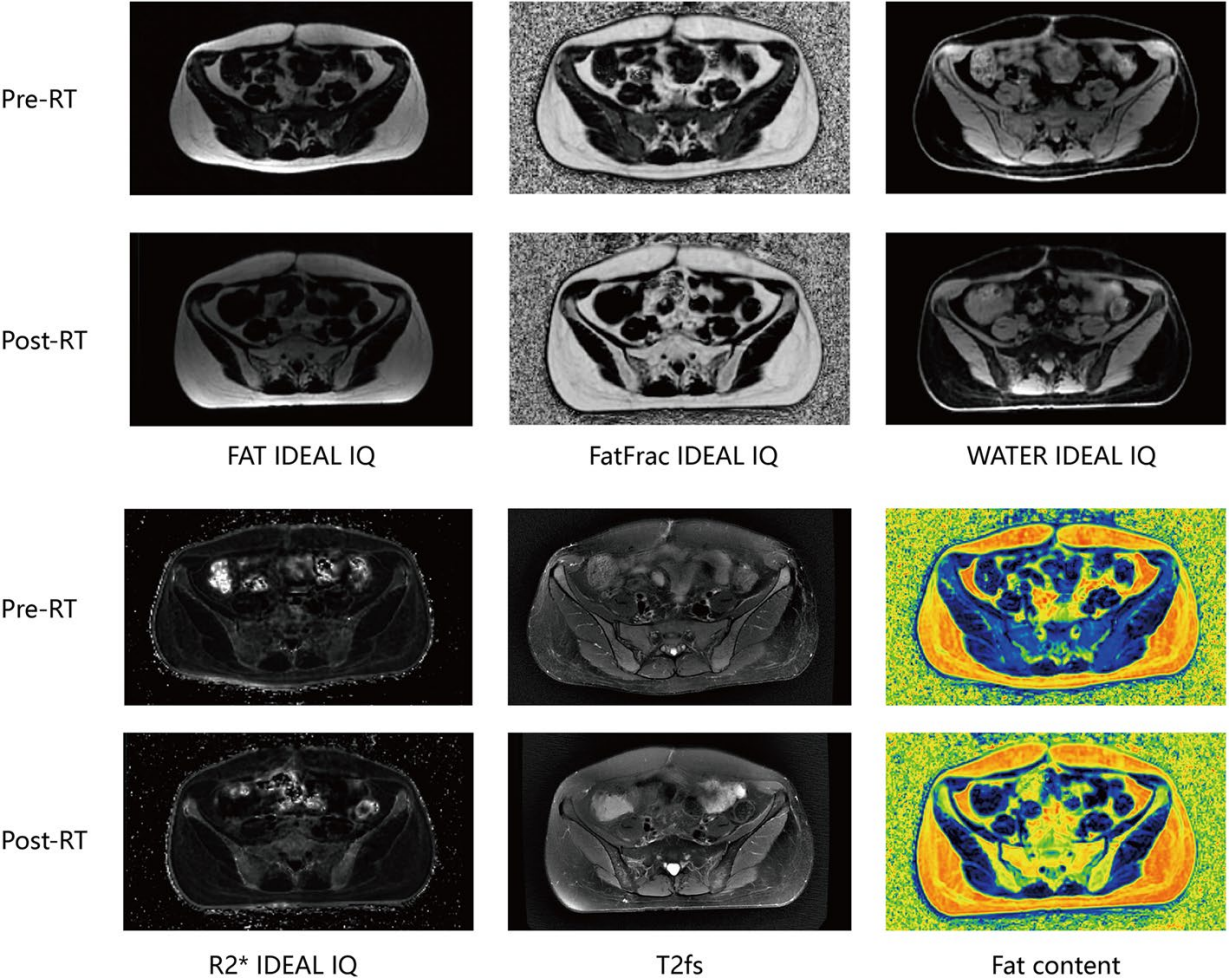
MR scan images of the same patient before and after radiotherapy

All MRI acquisitions were performed on a 3.0-T MRI system (Discovery MR 750, GE Medical System) with the same position and fixed device as CT scans. All patients underwent T2fs and IDEAL IQ serial scans (detailed MR sequences are listed in Supplementary Table 1). Patients underwent MRI with the same sequence at pre-RT (before the treatment) and post-RT (after the treatment). The IDEAL IQ scan was reconstructed to obtain four phases of the image, namely FatFrac IDEAL IQ, R2* IDEAL IQ, WATER IDEAL IQ, and FAT IDEAL IQ (Fig. 1).

### Radiation contouring and planning

The CT and MR images were imported into Eclipse15.5 (Varian, USA) planning system for tumor and organ at risk (OAR) contouring. The clinical target volume (CTV) included the gross tumor volume (GTV), the uterus, cervix, parametrium, upper third of the vagina, and the locoregional lymph nodes, which included the common, internal and external iliac, obturator, and presacral lymph nodes. The corresponding planning target volume (PTV) was generated from the CTV with 5 mm margins. The OARs included the bladder, rectum, small intestine, spinal cord, and delineated the bone marrow cavity from the lower edge of the L4 vertebral to the end of the femoral heads as PBM. The IMRT or VMAT plan used 6 MV photon beams, and the standard for the acceptance of the plan was that at least 95% volume of the PTV received 100% of the prescription dose. All patients underwent IMRT or VMAT to 45.0-50.4 Gy in 25–28 daily fractions to the PTV.

### Regions of interest (ROI) identification

**Fig. 2:**
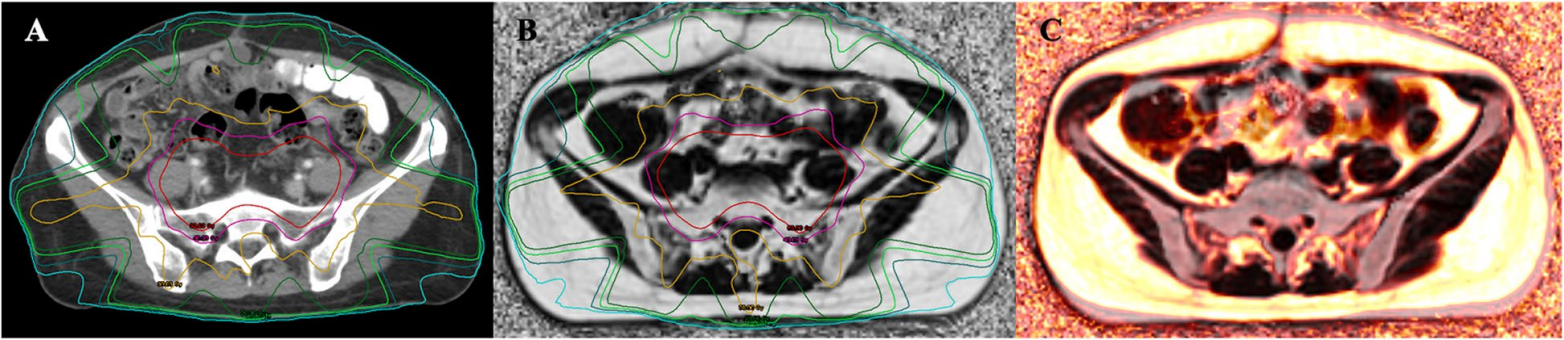
The schematic diagram of the dose map and rigid registration. Radiation dose distribution on a CT image (**A**); Radiation dose distribution on a MR FatFrac image (**B**); Fusion of the pre- and post- MR rigid registered images (**C**)

Based on the actual radiation dose of each patient, dose maps were generated in the Eclipse treatment planning system. According to the irradiated dose from low to high, it was divided into 5-10 Gy, 10-15 Gy, 15-20 Gy, 20-30 Gy, 30-40 Gy, 40-50 Gy and > 50 Gy dose regions, and then the corresponding 7 dose gradients were generated, reffered to the setting used by Chen et al. [16]. Dose maps, CT and MR scanning images, and RT plans were transferred to the MIM Maestro (version 6.8.2, USA). The CT images were rigidly registered with all the pre-RT and post-RT MR images, ensuring the consistency of the spatial coordinates and the shape of the two images. Cortical bone was used as the primary registration criterion. We manually adjusted the registered images if the registration error was greater than 1 mm. The displacement vector field was applied to the three-dimensional dose distribution related to the planning CT. The dose distribution in the bone marrow was obtained directly from the MRI images (Fig. 2).

The ROIs were manually delineated in the iliac bone (excluding cortical edges or blood vessels) under different dose gradients on pre-T2fs images; the volume of a single ROI under each dose gradient was ≥ 0.5 cm^3^. To ensure the consistency of ROI positions at different times and different sequences, T2fs images with delineated ROIs were rigidly registered with other MR images pre- and post-RT. The ROI positions were manually modified if necessary. All ROIs were obtained at the same position under different dose gradients on MR fused images pre- and post- radiotherapy to ensure the consistency of the spatial coordinates and the shape of ROIs.

### Texture features extraction, selection, and analysis

RF extraction was performed using 3D Slicer version 4.11. Before feature extraction, we used a fixed bin width of 25 to discretize the images. Ninety-three features were extracted from each ROIs of both T2fs and IDEAL IQ images. Eighteen firstorder texture features extracted from the tumor intensity histogram reflected the distribution of the values of individual voxels without concern for spatial relationships. Seventy-five texture features described the spatial arrangement of voxels and were calculated from different parent matrices. The matrices included the grey level cooccurrence matrix (GLCM), the grey level run length size zone matrix (GLRLM), grey level size zone matrix (GLSZM), grey level dependence matrix (GLDM), and the neighborhood grey-tone difference matrix (NGTDM).

A one-way analysis of variance (ANOVA) was applied for pre-RT RFs under each gradient, and the LSD test was used to verify the differences between groups under different dose gradients. RFs with a *p*-value of > 0.05 were retained to ensure consistency in feature extraction under each gradient. A paired-samples t-test and Wilcoxon signed rank test were performed for RFs with delineation consistency to select RFs with significant differences in pre and post-RT under different gradients in each image. A *p*-value of < 0.05 was considered statistically significant. Furthermore, common RFs were selected under all dose gradients.

**Fig. 3:**
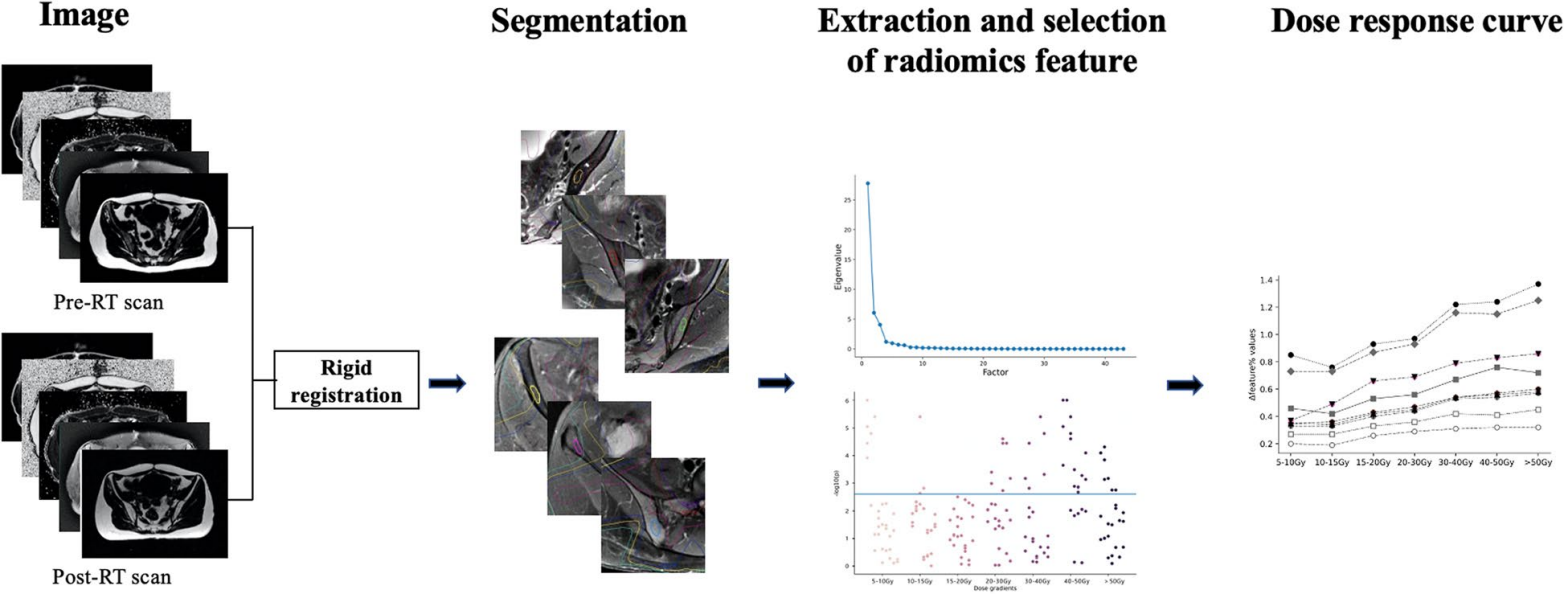
Summary of this workflow

Normalization was performed on the selected RFs. Principal component analysis (PCA) was performed using the normalized FAT IDEAL IQ radiomics features. PCA was an unsupervised data dimensionality reduction method that converted the relevant variables into principal components, which still contained most of the information from the original variables and helped determine the number of influencing factors. The first two or more components were selected, ensuring that at least 80% of the total variance was explained. The summary of this study workflow is shown in Fig. 3.

### Analysis of peripheral blood cell counts

PBC counts were analyzed weekly for all patients during radiotherapy. Absolute counts of white blood cells (WBC), neutrophils (ANC), lymphocytes (ALC), platelets (PLT), and hemoglobin (HGB) were prospectively collected at pre-RT, at midpoint RT, end-RT, and one month post-RT. The rate of change in PBC counts was measured before and after radiotherapy using the following formula: Δ count= $\frac{\text{c}\text{o}\text{u}\text{n}\text{t}\text{p}\text{o}\text{s}\text{t}-\text{c}\text{o}\text{u}\text{n}\text{t}\text{p}\text{r}\text{e}}{\text{c}\text{o}\text{u}\text{n}\text{t}\text{p}\text{r}\text{e}}$×100%. The nadir of PBC counts also reflects the degree of bone marrow injury. Therefore, we focused on changes in the PBC counts at the two time points before and at the end of radiotherapy to explore the factors that can reduce the absolute value of the nadir of PBC counts. To better provide an individualized bone marrow sparing program and reduce the decline in PBC counts.

### Statistical analysis

The effects of RT on bone marrow as the RF values change before and after radiotherapy were evaluated using the following formula: ΔRF: ΔRF= $\frac{\text{p}\text{o}\text{s}\text{t}\text{R}\text{T}-\text{p}\text{r}\text{e}\text{R}\text{T}}{\text{p}\text{r}\text{e}\text{R}\text{T}}$×100%. The dose-response curves were built considering the features calculated in the different isodose regions. Pearson’s correlation analysis was performed for ΔRF between changes in PBC counts. Data analysis was performed with IBM SPSS 26.0 (IBM SPSS Inc, Chicago, IL) and Python 3.8.8.

## Results

**Fig. 4:**
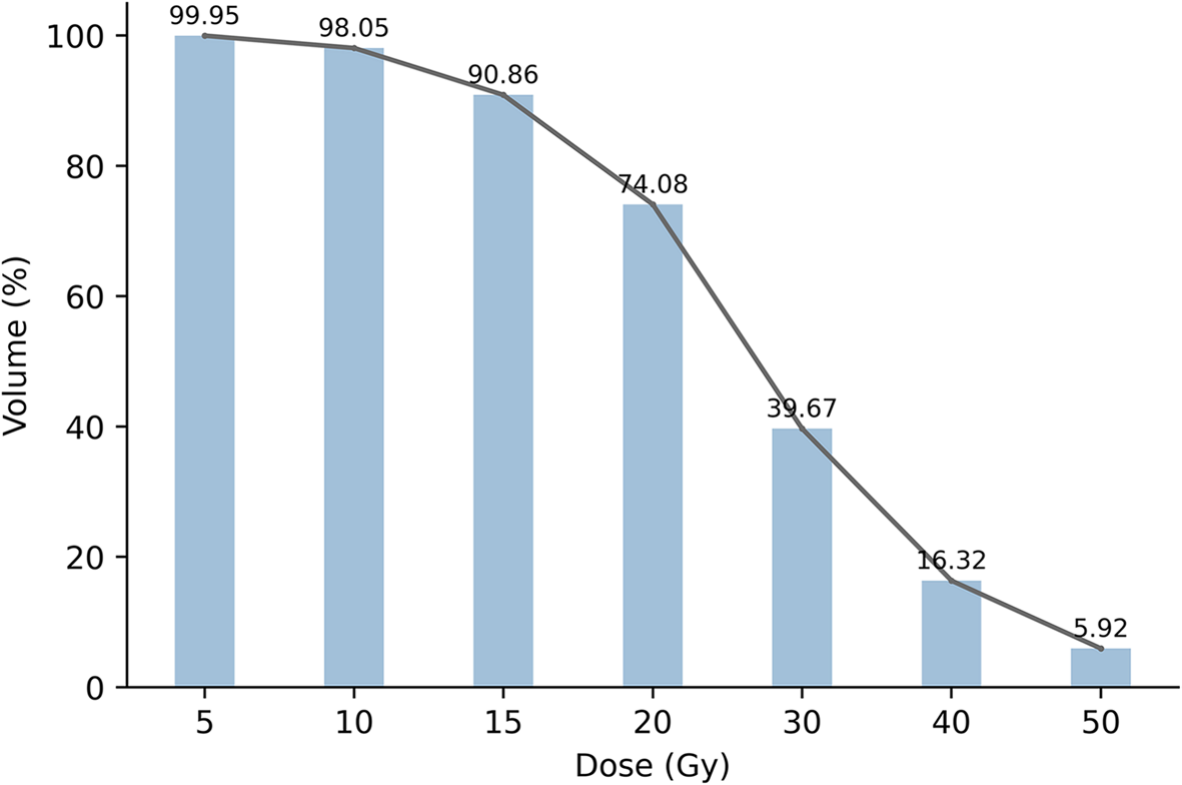
The dose-volume curve of ilium bone marrow

**Fig. 5:**
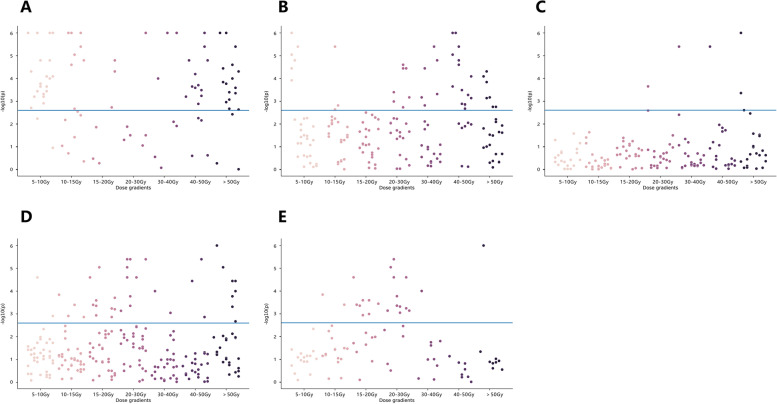
Manhattan plot of FAT IDEAL IQ (**A**), FatFrac IDEAL IQ (**B**), R2^*^ IDEAL IQ (**C**), WATER IDEAL IQ (**D**), and T2fs (**E**). Each point is corresponding to a feature and the blue line represents the *p* value equal to 0.05

The mean ilium bone marrow volume of all patients receiving 5 and 10 Gy irradiation accounted for 99.95% and 98.05% of the total bone marrow volume, respectively, while the mean ilium bone marrow volume receiving 50 Gy irradiation accounted for only 5.92% of the total bone marrow volume (Fig. 4). Among the RFs of different image sequences, the results of the univariate analysis are shown in Fig. 5. The characteristic distributions with statistically significant changes in RF before and after radiotherapy are shown in Table 2, which were inhomogeneous and mainly concentrated in the firstorder category.

**Table 2:** Number of features with statistically significant differences before and after radiotherapy under different sequences and dose gradients

FAT IDEAL IQ							
	Firstorder	GLCM	GLDM	GLRLM	GLSZM	NZTDM	Total
5-10 Gy	11	5	1	3	4	1	25
10-15 Gy	9	5	0	2	3	1	20
15-20 Gy	9	5	1	3	5	1	24
20-30 Gy	9	5	1	3	3	1	22
30-40 Gy	10	5	1	3	3	1	23
40-50 Gy	11	5	0	2	4	1	23
> 50 Gy	10	6	1	2	4	1	24
WATER IDEAL IQ							
	Firstorder	GLCM	GLDM	GLRLM	GLSZM	NZTDM	Total
5-10 Gy	0	1	0	1	0	0	2
10-15 Gy	1	1	1	0	1	1	4
15-20 Gy	5	1	1	2	1	1	11
20-30 Gy	6	2	1	0	0	1	10
30-40 Gy	8	1	1	1	0	0	11
40-50 Gy	8	1	2	4	1	0	16
> 50 Gy	8	1	2	4	1	0	16
FatFrac IDEAL IQ							
	Firstorder	GLCM	GLDM	GLRLM	GLSZM	NZTDM	Total
5-10 Gy	9	0	0	0	0	0	9
10-15 Gy	9	0	2	2	0	0	13
15-20 Gy	9	0	0	0	0	0	9
20-30 Gy	9	3	0	0	4	1	17
30-40 Gy	9	3	0	0	6	0	18
40-50 Gy	9	7	2	2	5	2	27
> 50 Gy	9	4	1	0	2	0	16
R2 IDEAL IQ							
	Firstorder	GLCM	GLDM	GLRLM	GLSZM	NZTDM	Total
5-10 Gy	0	0	0	0	0	0	0
10-15 Gy	0	0	0	0	0	0	0
15-20 Gy	0	0	0	0	0	0	0
20-30 Gy	1	0	0	0	0	0	1
30-40 Gy	1	0	0	0	0	0	1
40-50 Gy	2	0	0	0	0	0	2
> 50 Gy	2	1	0	0	0	0	3
T2fs							
	Firstorder	GLCM	GLDM	GLRLM	GLSZM	NZTDM	Total
5-10 Gy	0	0	0	0	0	0	0
10-15 Gy	1	0	1	0	0	0	2
15-20 Gy	5	2	1	0	0	1	9
20-30 Gy	6	3	2	0	0	1	12
30-40 Gy	8	0	1	0	0	0	9
40-50 Gy	8	0	1	0	0	0	9
> 50 Gy	8	0	1	0	0	0	9

### IDEAL IQ radiomics feature analysis

#### FAT IDEAL IQ radiomics feature

**Fig. 6:**
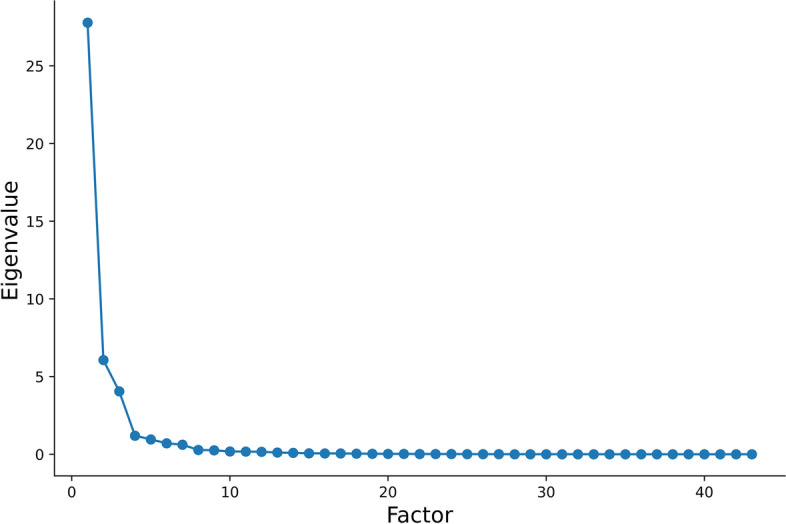
PCA scree plot. It displays the percentage of explained variances against the number of principal components

Most features were selected from FAT IDEAL IQ images with statistically significant differences before and after radiotherapy; the common features under each gradient were up to 19. Based on the PCA of common features extracted from the FAT IDEAL IQ sequences, the first two principal components explained 67.8% and 89.3% of the total variance, respectively, which explained most of the information on the common features (Fig. 6). The top five features that contributed to the first two principal components were all derived from the first order, which included 10Percentile, Mean, RootMeanSquared, Median, and Range.

**Fig. 7:**
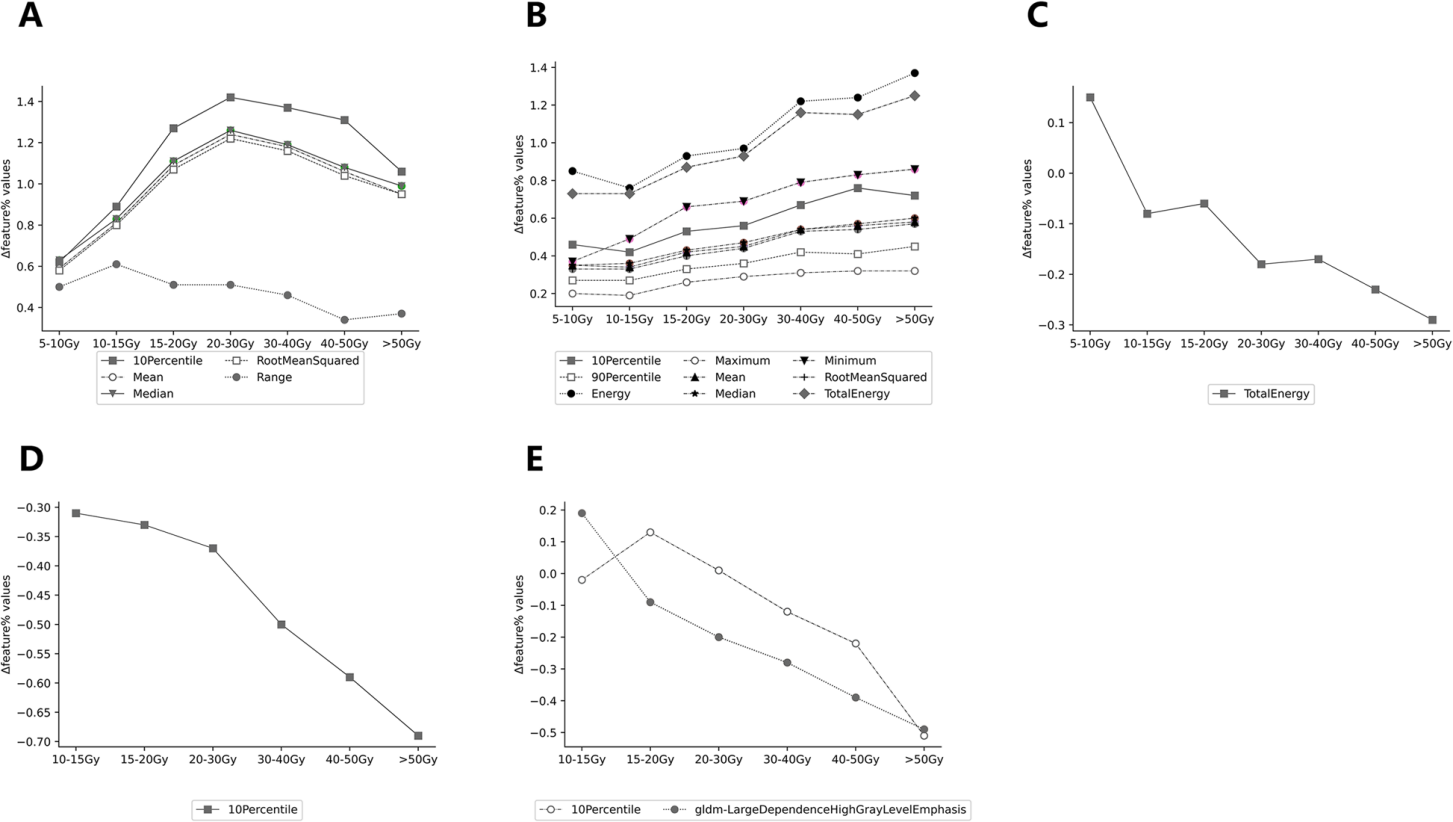
Dose-response curves of common characteristic in FAT IDEAL IQ (**A**), FatFrac IDEAL IQ (**B**), R2^*^ IDEAL IQ (**C**), WATER IDEAL IQ (**D**), and T2fs (**E**)

The rate of change in all the five features from the firstorder showed that the rate increased first and then decreased with the accumulation of dose. Except for Range, the other four features showed a larger change range and similar trends. The 10Percentile reached the maximum rate of change of 142.87% at 20-30 Gy. RootMeanSquared and Median showed a similar trend and the change rate ranged from 58.25%~125.63%. The magnitude and direction of the feature change varied widely between Range and the other features. The Range showed smaller changes with linear regression slopes of -3.60. (Fig. 7 A).

#### FatFrac IDEAL IQ radiomics feature

Under the seven different dose gradients in FatFrac IDEAL IQ images, the features with significant differences before and after radiotherapy were mainly concentrated in 40-50 Gy, which mostly belonged to the firstorder. Nine common features were selected under each dose gradient, all of which were firstorder features.

The changes in all common characteristics showed an increasing trend with dose accumulation. Firs-torder-Energy and firstorder-TotalEnergy varied widely (slope of 10.09 and 9.74, respectively), reaching the maximum rate of change of 125.02% and 137.25%, respectively, at > 50 Gy dose gradients. The trends and magnitudes of the other common features were basically the same, with a slope of 2.39 ~ 8.14 (Fig. 7B).

#### R2* IDEAL IQ radiomics feature

In the R2* IDEAL IQ images, more features were in the firstorder category with statistically significant differences before and after radiotherapy. One common feature was selected for all dose gradients, namely firstorder-Total Energy. Total Energy increased by 14.56% under the dose gradient of 5-10 Gy and showed a decreasing trend with the accumulation of radiation dose under > 10 Gy. The slope of the dose-response curve was − 6.12 and the maximum rate of change was − 29.28% (Fig. 7C).

#### WATER IDEAL IQ radiomics feature

Similar to the FatFrac IDEAL IQ image, significant features before and after radiotherapy in the WATER IDEAL IQ image belong mainly to the firstorder category, with the highest number of features in the > 40 Gy dose gradient. Among these features, a common feature was selected under the > 10 Gy dose gradients, namely firstorder-10Percentile. The change of 10Percentile showed a decreasing trend with the accumulation of radiation dose and reached the maximum change rate of -68.61% at > 50 Gy. The dose-response curve can be roughly fitted to a straight line with a slope of -7.93(Fig. 7D).

### T2fs radiomics feature analysis

In this analysis, no features were obtained with significant differences before and after radiotherapy under the 5-10 Gy dose gradient. The different features were mainly concentrated in the categories 15–20 Gy and firstorder categories. Two common features named firstorder-10Percentile and gldm-LargeDependenceHighGrayLevelEmphasis were obtained under > 10 Gy dose gradient. The direction of the two feature changes was different under 10-20 Gy. The overall trend of 10Percentile first increased and then decreased with dose accumulation, reaching the maximum change rate at 20 Gy (12.66%). The gldm-LargeDependenceHighGrayLevelEmphasis showed a downward trend with increasing dose, the dose-response curve with a slope of -12.54 (Fig. 7E).

### Correlations between the changes in radiomics features versus peripheral blood cell counts

**Fig. 8:**
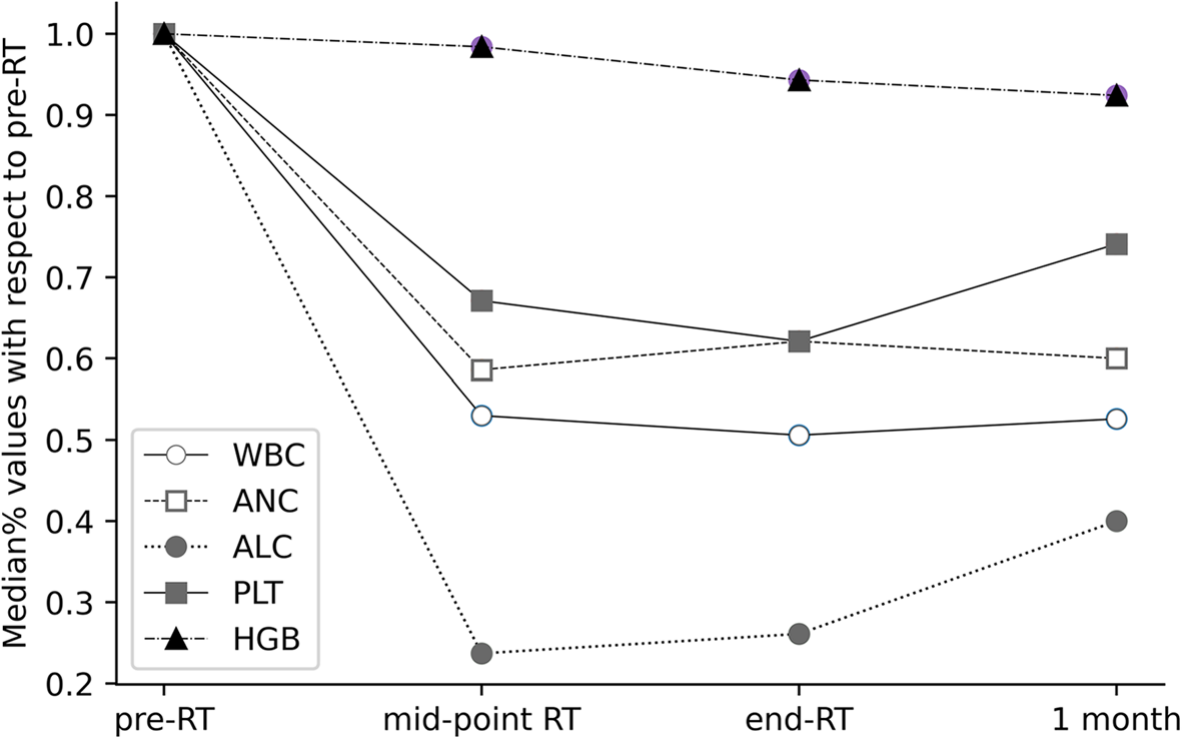
Overall trend of median white blood cell (WBC), neutrophil (ANC), lymphocyte (ALC), platelet (PLT) and hemoglobin (HGB) values with respect to pre-RT at different time intervals

The WBC, ANC, ALC, PLT, and HGB counts decreased significantly during treatment. The ANC and ALC nadirs occurred at the mid-point of radiotherapy (median values = 58.6% and 23.7% of pre-CCRT respectively), while the ANC and ALC nadirs occurred at the end of radiotherapy (median values = 50.6% and 62.1% of pre-CCRT respectively, Fig. 8).

Three common features were correlated with changes in PBC counts under each dose gradient of different sequences. Significant positive correlations were observed between Δfirstorder-Range and ΔANC-END under 5-10 Gy in FAT IDEAL IQ (*r* = 0.744, *p* = 0.001), indicating that increasing value of the firstorder-Range was correlated with decreasing ANC counts over the treatment course. Under 5-10 Gy dose gradient in T2fs, significant negative correlations were observed between Δfirstorder-10Percentile and ΔANC-END and ΔWBC-END (*r*=-0.654, *p* = 0.004; *r*=-0.563, *p* = 0.019). Similarly, significant negative correlations were observed between Δgldm-LargeDependenceHighGrayLevelEmphasis and ΔANC-END under 5-10 Gy in T2fs (*r*=-0.623, *p* = 0.01), indicating that the decrease in the firstorder-Range value was correlated with decreasing ANC counts over the treatment course.

## Discussion

The 3-year disease-free survival rate of cervical cancer patients receiving CCRT is up to 40% (stage IVA)–75% (stage IIB) [17]. Radiation and chemotherapeutic drugs can cause damage to hematopoietic stem cells and affect their self-renewal, manifested as yellow adipose tissue infiltration, fat, and fibrosis. The replacement of the hematopoietic bone marrow results in a decrease in HSC reserves and possible long-term or permanent damage to the hematopoietic system [18]. The slopes of the dose-response curve of the FatFrac and WATER IDEAL IQ radiomic features were consistent but in opposite directions. This reflected changes in the water and fat content at the microscopic level during the transformation of the red-yellow bone marrow. In the correlation analysis, IDEAL IQ and T2fs showed consistent changes with the changes in PBC.

Bone marrow sparing (BMS) radiotherapy reduces G2 + HT by approximately 70%, particularly leukopenia and neutropenia [2]. BMS-IMRT could reduce the volume of bone marrow receiving high-dose irradiation while maintaining the target coverage, resulting in fewer HT and potentially limiting the suppressive effect on immune responses [19–21]. Some retrospective studies have reported that low-dose irradiation of PBM was significantly associated with HT events [22, 23]. Zhu [24] et al. reported that with the mean dose of PBM increasing by 1 Gy, ANC and WBC were reduced by 9.6/µL and 7.8/µL per week, respectively. McGuire [25] et al. used FLT-PET to identify active bone marrow during radiotherapy and showed that a radiation dose of 4 to 5 4 to 5 Gy resulted in a reduction of approximately 50% in FLT uptake. Furthermore, suppression of bone marrow activity was measurable up to 1 year after radiotherapy, especially in patients with active PBM who received radiation doses greater than 25 Gy. It is critical to assess the radiation response of bone marrow, as this can predict radiation-induced damage.

IDEAL IQ can accurately assess bone marrow fat content using water-fat separation scanning technology, which can evaluate bone marrow fat content and changes in the bone marrow microenvironment [26–28]. Radiomics can explain the occurrence, progress, and outcome of diseases or response to treatment through medical imaging information and quantify changes in normal tissues during treatment. We explored the radiomic features of the IDEAL IQ sequence to quantitatively characterize changes in PBM injury under different dose gradients before and after radiotherapy. We further analyzed the dose-response relationship of the features of the texture of the magnetic resonance. Most firstorder features showed a clear dose-response relationship before and after radiotherapy; the dose-dependent effects were more pronounced especially in the FatFrac IDEAL IQ and WATER IDEAL IQ images. Nyflot [29] et al. have shown that first- and second-order features have higher repeatability than higher-order features. FatFrac IDEAL IQ provides quantitative information on bone marrow composition and allows non-invasive monitoring of the spatial effects of radiotherapy and quantifying the degree of bone marrow damage [30]. In our previous study, we used FatFrac IDEAL IQ to quantitatively assess bone marrow fat content changes. We found that the fat content increased significantly after radiotherapy in patients with cervical cancer and continued to increase with dose accumulation [14]. In the present study, the radiomics features of FatFrac IDEAL IQ images showed similar trends, revealing variation in bone marrow at the microscopic level. An obvious linear relationship was observed between different dose gradients and firstorder features in WATER IDEAL IQ images. Changes in firstorder features were stable in each image, showing that IDEAL IQ sequences can help detect radiotherapy-induced changes in bone marrow after cervical cancer radiotherapy.

Several studies showed that stochastic variability in MR acquisition details and reconstruction could impact some texture features [31, 32]. In this study, we used the same MR scanner, MR acquisition parameters, and details to minimize the image variance and improve the accuracy and robustness of MR-based features. We assessed the spatial relationship between the local radiation dose and the changes in the features of MRI radiomics. Analyzing changes in bone marrow radiomics features under different dose gradients can better assess the local bone marrow dose-response relationship. Our previous study reported that bone marrow fat content continued to increase six months after radiation therapy in the bone marrow area irradiated with > 30 Gy, indicating that bone marrow injury caused by high-dose radiation is difficult to reverse or repair in a short time, which is consistent with McGuire’s findings [14, 25]. This present study found that in FAT, WATER, and R2* IDEAL IQ images, the absolute value of the rate of changes in common features increased with dose accumulation, and bone marrow changes were more significant in the area receiving high-dose irradiation. This finding is consistent with our previous results, indicating that the delayed effect caused by high-dose irradiation is more persistent and difficult to repair.

The age and immune status of different patients develop individual differences in bone marrow status and PBC counts before radiotherapy. Therefore, it is necessary to ensure the stability of the baseline PBC counts before radiation therapy. In this study, different patients’ baseline levels of PBC counts of different patients were taken into account to calculate the rate of changes in PBC counts before and after radiotherapy. Furthermore, a correlation analysis was performed with the changes in radiomic features to avoid the effect of individual differences. Our previous study reported that changes in bone marrow fat content were significantly correlated with the ANC and ALC nadirs under the 5-10 Gy dose gradient. The present study observed significant positive correlations between firstorder-10Percentile and firstorder-Range changes versus differences in ANC counts under the 5-10 Gy dose gradient. Radiomics could help detect bone marrow injury with greater precision during CCRT.

Although the observed changes in bone marrow fat content were more pronounced in the high-dose region because the dose of cervical cancer radiotherapy is generally 45–50.4 Gy, the high-dose irradiated bone marrow occupies a smaller volume in the entire pelvic bone marrow. The low-dose irradiated bone marrow has a wider range, and low-dose irradiation has a greater impact on all bone marrow in the entire pelvis, which is probably the main reason we only found a correlation between increasing bone marrow fat content and PBC counts in the low-dose region. It is consistent with the existing literature, suggesting that the conservation of low-dose PBM irradiation in BMS radiation therapy should be emphasized.

This is the first study to evaluate bone marrow changes before and after radiotherapy using radiomics, quantify bone marrow spatial structure changes, and analyze the correlation between a feature and PBC counts changes. The linear relationship between the firstorder features of IDEAL IQ and the dose can better highlight the changes in the bone marrow induced by radiation than other features, indicating quantitative relationships between the features of the bone marrow and radiomics. The correlation between radiomics and PBC counts indicates the possibility of predicting bone marrow injury after radiation. The sample size was small, and all patients were from a single center; no prediction model for PBM damage can be created, which is a limitation of this work. Large data sets should be used in future studies to confirm our findings and explore other dose-response relationships. It will provide the possibility of setting a reliable dose and fractionation of bone marrow radiation, predicting the risk of HT, and developing personalized interventions. We will also establish an HT prediction model based on changes in radiation dose and radiomics to develop individualized BMS radiotherapy and to assess the risk of radiation-induced bone marrow injury.

## Conclusion

In conclusion, functional MR radiomics features were significantly correlated with radiation-induced changes in bone marrow, indicating a significant dose-response relationship. A significant correlation was found between changes in radiomics features and PBC counts in the low-dose region. These findings showed the potential of radiomics to provide a quantitative and dynamic assessment of bone marrow injury and provide an objective reference for bone marrow sparing during cervical cancer radiotherapy.

## Supplementary Information

**Additional file 1: Supplementary Table 1.** Imaging parameters for MRI protocol.

## Data Availability

The datasets generated and/or analyzed during the current study are not publicly available because they involve hospital patient information. However, they are available from the corresponding author upon reasonable request (mooneve_211@163.com).

## Abbreviations

CCRT: Concurrent chemoradiotherapy
RT: Radiation therapy
HT: Hematological toxicity
PBM: Pelvic bone marrow
MRI: Magnetic resonance imaging
RF: Radiomics features
PBC: Peripheral blood cell
FIGO: International Federation of Gynecology and Obstetrics
IMRT: Intensity modulated radiotherapy
VMAT: Volumetric modulated arc therapy
CT: Computed tomography
OAR: Organ at risk
CTV: Clinical target volume
GTV: Gross tumor volume
PTV: Planning target volume
ROI: Regions of interest
GLCM: Grey level cooccurrence matrix
GLRLM: Grey level run length size zone matrix
GLSZM: Grey level size zone matrix
GLDM: Grey level dependence matrix
NGTDM: Neighborhood grey-tone difference matrix
ANOVA: A one-way analysis of variance
PCA: Principal component analysis
WBC: White blood cells
ANC: Neutrophils
ALC: Lymphocytes
PLT: Platelets
HGB: Hemoglobin
BMS: Bone marrow sparing.
