# Supplementary material for: Functional MRI radiomics-based assessment of pelvic bone marrow changes after concurrent chemoradiotherapy for cervical cancer
Source: BMC Cancer. 2022 Nov 8;22:1149. doi: 10.1186/s12885-022-10254-7 (PMC9644624; doi:10.1186/s12885-022-10254-7)
Supplement: Supplementary file 1 — Additional file 1: Supplementary Table 1. Imaging parameters for MRI protocol. [file 12885_2022_10254_MOESM1_ESM.docx]

**Supplementary Table 1.** Imaging parameters for MRI protocol

| **Parameters** | **TR (ms)** | **TE (ms)** | **Field of view (cm)** | **Acquisition matrix**  **(phase × frequency)** | **Slice thickness**  **(mm)** | **NEX** | **Bandwidth** | **Acquisition time** |
| --- | --- | --- | --- | --- | --- | --- | --- | --- |
| T2fs | 11593 | 96 | 36 | 352 × 352 | 3.0 | 3.0 | 83.333 | 7.21 (min) |
| IDEAL IQ | 7.6 | Min full | 40 | 180 × 180 | 3.0 | 2.0 | 83.33 | 2.19 (min) |
